# LC-MS/MS of PEth in whole blood: implementation in routine clinical chemistry

**DOI:** 10.1016/j.plabm.2026.e00533

**Published:** 2026-04-21

**Authors:** Jakob Albrethsen, Ditte Hermann, Ninna Hahn Tougaard, Sofie Boesgaard Neestrup Hansen, Nina Kimer, Lise Lotte Gluud, Nicolai J. Wewer Albrechtsen

**Affiliations:** a Department of Clinical Biochemistry, Copenhagen University Hospital – Bispebjerg and Frederiksberg, Copenhagen, Denmark; b Department of Clinical Medicine, Faculty and Medical Sciences, University of Copenhagen, Copenhagen, Denmark; c Gastrounit, Copenhagen University Hospital – Hvidovre, Hvidovre, Denmark

**Keywords:** PEth, LC-MS/MS, Whole blood

## Abstract

In clinical practice, assessing alcohol intake can be challenging because patients may under-report or misjudge their consumption, so biomarkers of chronic intake provide valuable additional information. Phosphatidylethanol (PEth) is a group of abnormal phospholipids that are formed in cell membranes only in the presence of ethanol (alcohol). We developed, analytically validated, and clinically implemented a rapid and robust LC-MS/MS-based method for quantifying PEth (16:0/18:1) in whole blood. The method demonstrates a limit of detection (LOD) of 0.01 μM and a limit of quantification (LOQ) of 0.03 μM, with confirmed linearity up to at least 0.8 μM. Validation using reference materials and samples from patients with liver disease and self-reported alcohol intake confirmed its accuracy. Clinical implementation over a two-month period, involving 20 analytical batches, demonstrated inter-assay precision below 15%. Our results support the clinical applicability of PEth use for accurate estimation of alcohol intake in humans.

## 1 Introduction

The World Health Organization estimates that 2.8 million deaths annually (i.e., 4.7% of all deaths) are caused by alcohol consumption [1]. A considerable proportion of patients with alcohol use disorder (AUD) exhibit discrepancies between their self-reported alcohol consumption and objective biomarker results [2]. This inconsistency is mainly due to under-reporting and highlights the limitations of relying solely on patient self-report for assessing alcohol use. Phosphatidylethanol (PEth) is a biomarker for alcohol intake and is of increasing clinical importance due to its relatively long detection window [3,4].

PEth in whole blood complements traditional biochemical markers for alcohol intake since it can detect moderate to heavy alcohol intake up to four weeks prior to sample collection [5]. Clinicians often rely on questionnaires to collect information about alcohol consumption, though self-reporting can be misevaluated or undermined by memory bias, under-reporting and stigma [6]. Accordingly, biochemical markers are an important supplement for detecting and classifying unhealthy alcohol intake. Several markers for alcohol consumption are used today. Testing for breath alcohol concentration or blood alcohol concentration can detect very recent alcohol intake (up to ∼24 h). Other traditional alcohol biomarkers in blood and urine, such as ethyl glucuronide and ethyl sulfate, can detect alcohol consumption up to five days prior to sample collection. In addition, measurement of liver enzymes is used to monitor the toxic effect of ethanol and for the identification of heavy drinkers but are not sufficiently sensitive to detect moderate alcohol consumption and are not specific for alcohol misuse. PEth is a group of phospholipids that are formed in the body after alcohol intake [7,8]. The normal function of the enzyme phospholipase D (PLD) is to convert phosphatidylcholine into phosphatidic acid (PA) and choline. In the presence of ethanol, PLD incorporates ethanol into the phospholipid structure, resulting in the formation of PEth [9]. Thus, PEth is specific for alcohol intake. Secondly, after formation, PEth inserts into the cell membrane of red blood cells and is detectable in whole blood for several weeks after alcohol consumption [10]. Several PEth isoforms are formed in humans after alcohol intake [7,8]. The isoform known as PEth (16:0/18:1) is the most abundant and is commonly used for clinical PEth measurement [3]. So far, findings support PEth screening in liver disease [[11], [12], [13]] and additional clinical utilities of PEth measurement are being explored [14].

Here we develop and validate an LC-MS/MS method for PEth (16:0/18:1) in whole blood. We adopted the “Swedish criteria” for clinical PEth measurement in whole blood [15]. According to these criteria a whole blood concentration of PEth (16:0/18:1) less than 0.05 μM indicates no or low alcohol consumption, whereas a concentration of 0.05–0.30 μM PEth indicates a moderate intake of alcohol. A PEth concentration higher than 0.30 μM indicates a substantial and regular use of alcohol. Therefore, the goal was to develop a method with LOQ below 0.05 μM, a linear response to well above 0.05 μM and the inter-assay imprecision should be acceptable (<20%). The presented method is based on previously published protocols [16,17]. Our emphasis was on establishing a fast and cheap sample extraction procedure suited for PEth measurements in a routine setting.

## 2 Methods and materials

### 2.1 Participants

The first cohort of participants included anonymous volunteers at the Department of Clinical Biochemistry, Bispebjerg Hospital. Ethical approval was provided by the local ethical committee. Participants were given informed written consent prior to sampling and were asked to report their units of alcohol consumption in the previous 4 weeks. Inclusion criteria were either self-reported abstinence or low to moderate alcohol intake. One unit of alcohol was defined as one glass of wine or one beer.

The second cohort of participants was recruited from a prospective cohort of patients diagnosed with alcohol related liver disease within three months prior to sampling. The study is approved by the Regional Scientific Ethics committee (jr. no: H-24053649) and registered in clinicaltrials.gov (NCT06866496). All participants were recruited from Hvidovre University Hospital and gave informed written consent prior to sampling. Samples were collected between June 19 and September 18, 2025. Information on alcohol consumption was self-reported as AUDIT-C, and Timeline Follow-back was conducted with a specially trained hepatology nurse at the same day of blood sampling.

The third cohort of participants was recruited from a cross-sectional cohort of patients diagnosed with metabolic dysfunction-associated steatotic liver disease (MASLD) and gall stone disease. The study is approved by the Regional Health Research Ethics committee (H-20047626) and Data Protection Agency (P-2021-187). All participants have been recruited from Hvidovre University Hospital and given informed written consent prior to sampling. Samples have been collected between June 2022 and September 2024. Alcohol consumption was self-reported as AUDIT-C.

Study participants from cohort 2 and 3 underwent a diagnostic liver biopsy according to the best clinical practice. The scoring of the liver tissue was made by trained pathologist. The participants in cohort 2 were categorized by fibrosis scoring (F-stages) and cirrhosis. For a subset of individuals, these participants also had autoimmune hepatitis (AH). The study participants were categorized in cohort 2 based on having normal liver tissue, mild steatosis or MASH.

Finally, 8 external quality PEth (16:0/18:1) control samples were obtained from Equalis (Article number: 295, Uppsala, Sweden) and prepared according to the manufacturer's instructions.

### 2.2 Collection of whole blood samples

Whole blood was sampled from a peripheral vein and was collected in EDTA tubes (BD, K2EDTA, 455045) that were not centrifuged. All samples were stored at −80 °C for ≥20 h before analysis to ensure hemolysis and were immediately refrozen at −80 °C after use. All Samples were stored for maximum two months at −80 °C before analysis. Samples were turned for equal distribution before pipetting.

### 2.3 Chemicals

All solvents and reagents—LC-MS-grade methanol (MeOH PN:34966), isopropanol (IPA, PN:34965), heptane (HEP PN:34873), acetonitrile (ACN PN:34967), and ammonium acetate (AA PN:102869012)—were obtained from Sigma-Aldrich (Denmark). LC-MS-grade deionized water was sourced from a Purelab Flex dispenser (ELGA LabWater, UK).

### 2.4 Preparation of PEth standards and quality controls

A PEth standard stock solution (25 mg PEth (16:0/18:1) in 2.5 mL chloroform 840514C-25 mg Avanti, US) was diluted in 7.5 mL organic solvent (50% IPA, 50% ACN). A PEth-D5 internal standard stock solution (1 mg PEth-D5 (16:0/18:1) in 1.0 mL methanol, P-164-1ml, Supelco, US), was diluted in in 9.0 mL organic solvent (50% IPA, 50% ACN). Standards and quality control (QC) samples were prepared using in-house collected blank whole blood from alcohol-abstinent volunteers, verified to be PEth-free by triplicate LC-MS/MS analysis. Four calibration standards were prepared by spiking blank whole blood with the PEth stock solution to achieve the following concentrations: C1: 0.026 μM, C2: 0.048 μM, C3: 0.175 μM and C4: 0.819 μM. Three QC samples were prepared. QC1 (0.020 μM) and QC2 (0.065 μM) were prepared by spiking blank whole blood with PEth. QC3 (0.110 μM) was prepared by spiking a pool of PEth-positive whole blood samples with the PEth stock solution.

### 2.5 Extraction procedure

The extraction solution was prepared by adding 10 μL of PEth-D5 stock to 8 mL of organic solvent mixture (37.5% HEP, 25% MeOH, 37.5% IPA). Uncentrifuged whole blood samples (patient, volunteer, calibrant, and QC samples) were thawed at room temperature for ∼20 min after retrieval from −80 °C storage. Samples were then placed on a rocker (Phoenix R5-TRO5) for at least 5 min before pipetting. For PEth extraction, 20 μL of whole blood was transferred to Eppendorf Low Bind tubes (Eppendorf, 022431021), followed by the addition of 120 μL of extraction solution. The tubes were shaken (Eppendorf MixMate 5353, 1900 rpm, 30 min, room temperature) and centrifuged (Eppendorf centrifuge 5417R, 16,400×*g*, 5 min, room temperature). The supernatant (120 μL) was transferred to 96-well plates (Nunc MicroWell) for LC-MS/MS analysis. After processing, the remaining blood samples were immediately refrozen at −80 °C.

### 2.6 LC-MS/MS analysis

Samples were analyzed using an Acquity UPLC system (Waters, Milford) coupled to a TQ-S triple quadrupole mass spectrometer (Waters). A 10 μL aliquot of each sample was injected onto a BEH C18 column (1.7 μm, 2.1 × 100 mm, Waters). Analytes were separated over a 7-min gradient at a flow rate of 0.3 mL/min, using Solvent A (50 mM ammonium acetate) and Solvent B (90% MeOH, 10% IPA). The gradient started at 70% B, increased to 95% B, followed by a 2-min flush at 100% B, and two brief washes (shifting between 100% A and 95% B) to eliminate carry-over before returning to initial conditions. Mass spectrometry was performed in negative ion electrospray ionization mode, with the following settings: Capillary voltage: 3 kV, Source gas flow rate: 150 L/h, Collision gas flow rate: 0.15 mL/min, Collision energy: 38 V, Cone voltage: 50 V, Dwell time: 50 ms per ion. Selected reaction monitoring (SRM) transitions for PEth and PEth-D5 are detailed in Table 1. Spectral data were processed using TargetLynx XS (v4.1, Waters).

**Table 1:** MS acquisition parameters.

Class	Analyte	Precursor Ion (*m/z*)	Product Ion (*m/z*)	Collision Energy (eV)
Quantifier	PEth 16:0/18:1	701.6	255.2	38
Quantifier-internal standard	PEth-IS 16:0/18:1:D5	706.6	255.2	38
Qualifier	PEth 16:0/18:1	701.6	281.2	38
Qualifier-internal standard	PEth-IS 16:0/18:1:D5	706.6	281.2	38

### 2.7 Data analysis

Statistical analyses, including mean, standard deviation (SD), coefficient of variation (CV), and R^2^ values, were performed using Microsoft Excel (2016). The significance of correlations was assessed using the Correlation function in Excel's Analysis ToolPak. The limit of detection (LOD) and limit of quantification (LOQ) were calculated as follows: LOD = 3.3 × (σ/S) & LOQ = 10 × (σ/S), where σ represents the standard deviation of three blank samples measured over three different days, and S is the slope of three calibration series measured on three separate days.

## 3 Results

### 3.1 Development of a PEth LC-MS/MS assay

**Fig. 1:**
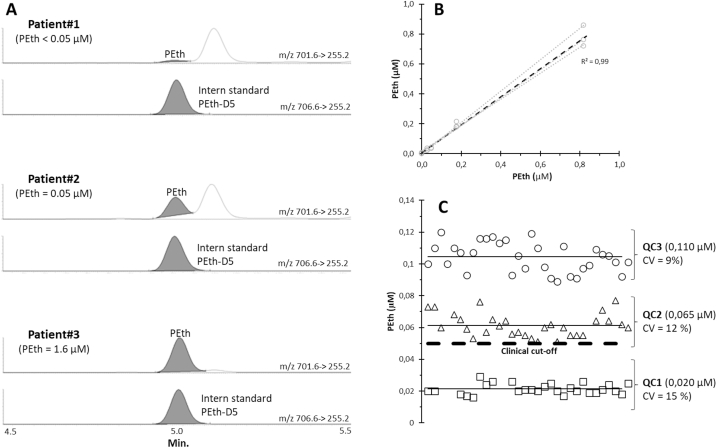
LC-MS/MS analysis of whole blood phosphatidylethanol (PEth). **A** Representative LC-MS/MS chromatograms of PEth and its deuterium-labeled internal standard (PEth-D5) from three patients with whole blood PEth concentrations of <0.05 μM, 0.05 μM, and 1.6 μM, respectively. **B** PEth calibrant series measured across three independent batches (gray dotted lines), with the average calibration curve represented by the black dotted line. **C** Longitudinal precision of PEth quality controls (QCs 1–3) evaluated in duplicate over 20 batches during a three-month period, yielding coefficients of variation (CVs) of 15%, 12%, and 9%, respectively.

To optimize sample preparation and LC-MS/MS conditions, a pool of blank whole blood from local volunteers was spiked with PEth and the deuterated internal standard (PEth-D5) was added to the different extraction solutions. After evaluating extraction protocols, a single-step extraction method was selected for its efficiency and reproducibility: 20 μL of whole blood was diluted in 120 μL of a solvent mixture containing 37.5% heptane, 25% methanol, and 37.5% isopropanol. This simple protocol provided a reliable and reproducible signal and deemed suitable for a routine workflow. For LC-MS/MS analysis, the two most sensitive and selective transitions for PEth were evaluated (Table 1). The *m/z* 701.6 > 255.2 transition was chosen as the quantifier ion, while the *m/z* 701.6 > 281.2 transition served as the qualifier ion for confirmation (Fig. 1A).

### 3.2 Analytical validation of the PEth LC-MS/MS assay

Calibration curves, prepared and measured across three independent days, demonstrated linearity across the range from the limit of detection (LOD) to 0.8 μM, with consistent overlap between runs (Fig. 1B). The LOD of the method was determined from the calibration data, yielding an LOD of 0.01 μM and an LOQ of 0.03 μM (Table 1). To assess the accuracy, eight external PEth quality control (QC) samples (0.066–1.3 μM) from Equalis (Uppsala, Sweden) were analyzed. The average bias was +8.4% (range: 2.7–13.8%) (Table 2). Carry-over was investigated by injecting samples with high concentrations of PEth (6 μM) followed by three subsequent blank samples. Carry-over was minimal (<0.1%) and effectively eliminated by incorporating a second column flush with a high concentration of organic solvent after each run. Finally, the PEth method was introduced into routine analysis for three months and the inter-assay imprecision was <15% in 20 batches performed over two months (Fig. 1C).

**Table 2:** External quality control.

PEth reference material (μM)	PEth measured (μM)	Difference (%)
**0,060**	**0,066**	**10,2**
**0,288**	**0,300**	**4,1**
**1201**	**1336**	**10,1**
**0,387**	**0,447**	**13,4**
**0,695**	**0,806**	**13,8**
**0,000**	**0,000**	**0,0**
**0,300**	**0,310**	**2,7**
**0,660**	**0,750**	**12,8**
	**Mean diff.(%)**	**8,4**

### 3.3 Clinical validation of the PEth LC-MS/MS assay

**Fig. 2:**
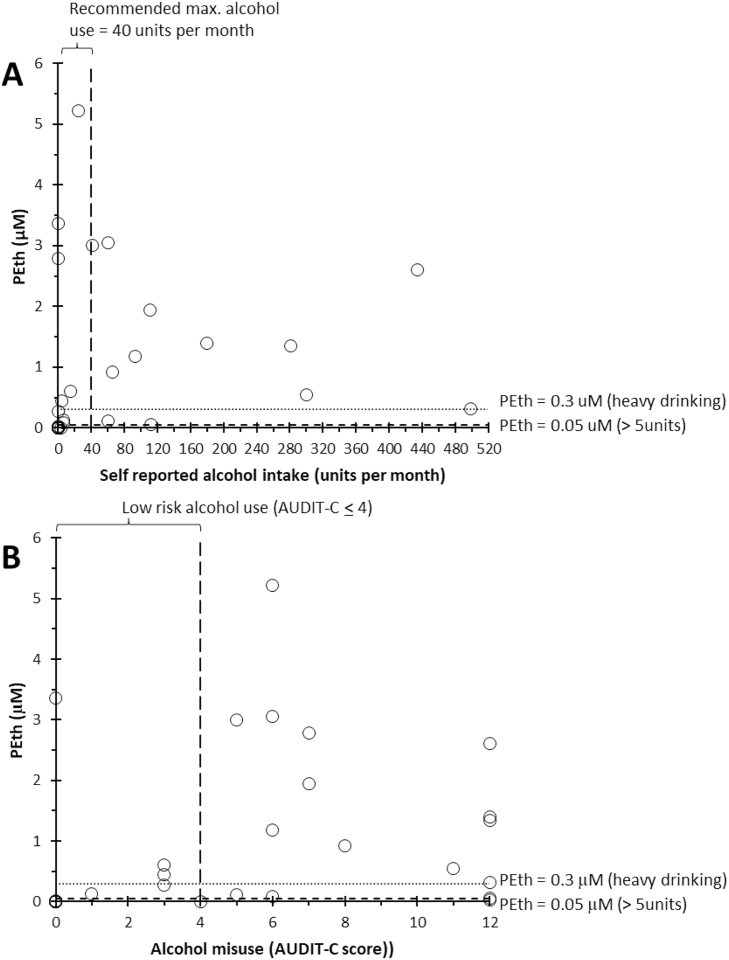
LC-MS/MS analysis of whole blood phosphatidylethanol (PEth) in 32 patients with alcohol-induced liver disease (see Table 4 for patient information). **A** Correlation between self-reported alcohol intake (alcoholic units per month) and PEth concentration (μM). Horizontal dotted lines indicate thresholds for positive alcohol intake (PEth >0.05 μM) and heavy drinking (PEth >0.3 μM). The vertical dotted line represents the maximum recommended monthly alcohol intake according to the Danish Health Authority (40 alcoholic units per month). **B** Relationship between the degree of alcohol misuse, assessed by the Alcohol Use Disorders Identification Test (AUDIT-C) score, and PEth concentration (μM). Horizontal dotted lines indicate thresholds for positive alcohol intake (PEth >0.05 μM) and heavy drinking (PEth >0.3 μM). The vertical dotted line denotes the low-risk alcohol intake range (AUDIT-C score: 0 to 4).

**Fig. 3:**
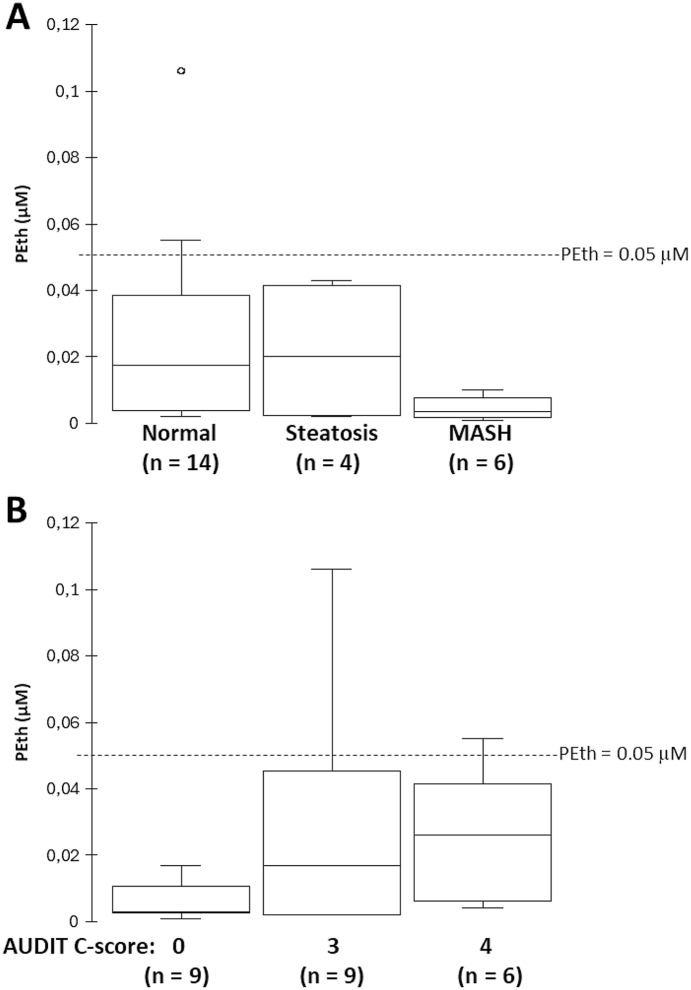
LC-MS/MS analysis of whole blood phosphatidylethanol (PEth) in 10 patients with liver disease and 14 healthy subjects (see Table 5 for details). **A.** Whole blood PEth concentrations (μM) stratified by liver state: normal, steatosis, and metabolic dysfunction-associated steatohepatitis (MASH). The horizontal dotted line indicates the threshold for positive alcohol intake (PEth >0.05 μM). **B.** PEth concentration across the degree of alcohol misuse assessed by the Alcohol Use Disorders Identification Test (AUDIT-C) score, and PEth concentration (μM). The horizontal dotted line indicates the threshold for positive alcohol intake (PEth >0.05 μM).

PEth was measured in whole blood collected from three cohorts. The first cohort included 11 anonymized volunteers (Table 3). The concentration of PEth was <0.05 μM indicating no or very low alcohol intake in the eight volunteers with a self-reported alcohol intake between 0 and 5 units of alcohol in the 4 weeks before sample collection. The remaining three volunteers reported analcohol consumption between 5 and 12 units in the previous 4 weeks and here PEth concentration was between 0.06 and 0.12 μM indicating low to moderate alcohol intake. The second cohort included 32 patients with alcohol related liver disease and with highly divergent self-reported monthly alcohol intake (range: 0 - 499 units per month) (Table 4, Fig. 2). In this cohort, the concentration of PEth was >0.3 μM for 17 patients indicating high, regular alcohol consumption, PEth was between 0.05 and 0.3 μM in five patients indicating low to moderate alcohol consumption and PEth was <0.05 μM in ten patients indicating no or very low alcohol consumption. Overall, there was no significant correlation between PEth and the self-reported alcohol intake (Fig. 2A) or the Alcohol Use Disorders Identification Test (AUDIT-C)-score (range: 0 to 12) in the cohort of 32 liver patients (Fig. 2B). The third cohort included 10 healthy individuals and 14 patients with MASLD (Table 5, Fig. 3), all with low self-reported alcohol intake. The concentration of PEth was <0.05 μM in 21 of the individuals indicating no or very low alcohol intake and between 0.05 and 0.1 μM in three of the individuals indicating low to moderate alcohol intake. Furthermore, PEth concentrations were reported for subgroups of cohort three based on liver condition, including normal, mild steatosis or Metabolic dysfunction-Associated SteatoHepatitis (MASH), and subgroups based on the AUDIT C-score (range: 0 to 4) ((Fig. 3A) and B, respectively).

**Table 3:** Self-reported alcohol intake and PEth concentration for individuals in cohort 1.

Donor	Self-reported alcohol intake during previous 4 weeks (units per week)	PEth (uM)
1	0	<0.01
2	0	<0.01
3	0	<0.01
4	0	<0.01
5	0	<0.01
6	0-5	0.02
7	0-5	0.02
8	0-5	<0.01
9	5	0.07
10	5-10	0.06
11	12	0.12

**Table 4:** Clinical data, AUDIT C-score, self-reported alcohol intake and PEth concentration for individuals in cohort 2.

Age	Sex	Liver state	AUDIT C-score	Units_30_days	PEth (μM)
68	M	F3	7	0	2783
61	F	F3	5	60	0,108
57	M	Cirrhosis	0	0	0
67	F	Cirrhosis	0	0	0
70	M	Cirrhosis	4	3	0
67	M	F3	1	7	0,125
78	M	Cirrhosis	0	0	0
54	M	Cirrhosis	0	0	0
65	F	Fibrosis	8	66	0,922
69	M	Cirrhosis	3	15	0,604
49	F	Cirrhosis	6	60	3,05
61	F	Clinical cirrhosis	5	41	2998
71	M	Cirrhosis	6	93	1183
67	F	F3	12	281	1344
72	M	Cirrhosis	0	0	0
71	M	F1			0,631
59	M	Clinical cirrhosis	12	0	0,027
61	F	Clinical cirrhosis	6	6	0,091
62	F	Normal	3	4	0,442
74	F	F3			0
46	M	Clinical cirrhosis	12	499	0,308
79	M	F3 minimum	0	0	0,007
78	M	Clinical cirrhosis	7	111	1938
55	M	Clinical cirrhosis	6	24	5214
42	F	No cirrhosis or active AH	3	0	0,277
54	M	Clinical cirrhosis	0	0	0
46	F	Clinical cirrhosis	0	0	3359
65	M	AH + Clinical cirrhosis	12	112	0,057
64	M	AH + Cirrhosis	12	180	1,39
51	M	AH + Clinical cirrhosis	11	300	0,545
35	F	AH	12	434	2605
58	F	AH + Clinical cirrhosis			0,233

**Table 5:** Clinical data, AUDIT C-score and PEth concentration for individuals in cohort 3. Metabolic dysfunction-Associated SteatoHepatitis (MASH).

Age	Sex	Liver state	AUDIT C-score	PEth (μM)
26	Female	Steatosis	0	0,003
54	Female	MASH	0	0,004
49	Female	Steatosis	0	0,002
55	Male	Normal	0	0,017
58	Female	Normal	0	0,003
43	Male	Steatosis	3	0,043
59	Female	Normal	3	0,106
67	Male	Normal	3	0,016
23	Male	MASH	0	0,001
72	Male	Normal	3	0,052
38	Female	Normal	4	0,024
38	Female	Normal	4	0,004
28	Female	Normal	3	0,002
30	Male	MASH	3	0,002
52	Male	MASH	4	0,007
63	Female	Normal	3	0,034
41	Female	Normal	4	0,028
40	Female	MASH	0	0,003
64	Male	MASH	0	0,01
44	Male	Normal	4	0,055
73	Female	Normal	3	0,002
41	Male	Normal	0	0,011
63	Male	Mild steatosis	4	0,037
63	Male	Normal	3	0,018

## 4 Discussion

We developed and validated an LC-MS/MS-based method for the quantification of phosphatidylethanol (PEth) in human whole blood. Clinically, PEth measurements in our laboratory are used to determine alcohol consumption, with a threshold of ≤0.05 μM indicating abstinence. Our validation demonstrates that the method meets this clinical and analytical requirement.

Previous studies have employed various methods for extracting PEth from whole blood, including different ratios of blood to organic solvent, diverse solvent combinations, and, in some cases, sequential extraction. We opted for a single-step extraction method. The LC-MS/MS settings were optimized based on previously reported protocols. In our experiments, the *m/z* 701.6 > 255.2 transition yielded the best performance for quantifying PEth in whole blood, aligning with findings from some studies [18]. However, most studies have favored the alternative PEth transition (*m/z* 701.6 > 281.2) for quantification (see for example 16). In our experience the optimal transition for PEth likely depends highly on the background interference, which is influenced by the method of extraction. We found that the LOD and LOQ of the method was 0.01 and 0.03 μM, respectively, and the method was linear in the concentration range up to 0.8 μM, at least. This agrees with most other published studies where the LOQ typically ranges from 0.002 to 0.05 μM [4,16]. We analyzed eight external QC samples from the Swedish national program for PEth measurement (Equalis, Sweden) and the average bias was +8.4%. This bias is within the range commonly observed in hospitals in Sweden [4,16]. The imprecision was determined using three QC samples with QC1 (0.02 μM) being just below the cut-off and QC2 (0.065 μM) just above, whereas QC3 (0.110 μM) reflects the imprecision of the high range. We find that the inter-assay precision of 20 batches analyzed for three months was below 15% for all three QCs which corresponds with recent findings [4,16]. In the present study we used in-house standards and QC samples, but commercial products may offer a more economical and practical alternative in the long term. In our laboratory we expect to analyze no more than 100 samples per week. In case of increased demand, it may be necessary to optimize the method, such as automation of pipetting steps, optimization of the extraction protocol and introducing a shorter analytical LC column for reduced time of analysis. A few Swedish hospitals currently measure >1000 samples per week and such high throughput would probably require switching to an automated method of sample preparation including solid phase extraction in a 96-well format [19]. Sample handling presents a unique challenge for PEth analysis in the routine setting. In our laboratory all blood samples for PEth analysis are stored locally at −80 °C for at least 20 h before analysis to ensure sufficient release of PEth from red blood cells. In addition, all samples, calibrants and QC samples are stored at −80 °C, although some studies have found that PEth is stable at 4 °C and −20 °C for up to 60 days [20].Our preliminary assessment of the clinical utility of PEth measurements across three small cohorts revealed a strong agreement between self-reported alcohol intake and PEth levels among individuals with low to moderate consumption. However, this correlation did not hold in a subgroup of 32 liver patients with highly divergent self-reported alcohol intake, where PEth concentrations suggested that several individuals either under- or over-estimated their alcohol consumption. This suggests that self-reported alcohol consumptions are unreliable. These findings also underscore the need for further research to clarify the clinical utility of PEth measurements, particularly given its inability to distinguish between recent short-term high-volume drinking and chronic problematic alcohol use [[21], [22], [23]]. Additionally, more studies are required to elucidate the factors influencing PEth formation and elimination, as well as to establish optimal clinical cut-off levels tailored to Danish populations. Future research should also evaluate the clinical and economic impact of integrating PEth measurement into routine analysis, including its potential to enhance patient outcomes and cost-effectiveness.

In conclusion, we find that the presented method for PEth measurement in whole blood is sufficiently sensitive, robust and cost-effective for routine analysis in a hospital setting. In future studies we will further examine the clinical utility of PEth. Presently, we propose that PEth could become useful in the subclassification of patients with liver disease, especially in patients with a history of high alcohol intake.

## Disclosure summary

The authors have nothing to disclose.

## Declaration statement

The work described has not been published previously except in the form of a preprint, an abstract, a published lecture, academic thesis or registered report.

The article is not under consideration for publication elsewhere.

The article's publication is approved by all authors and tacitly or explicitly by the responsible authorities where the work was carried out.

If accepted, the article will not be published elsewhere in the same form, in English or in any other language, including electronically, without the written consent of the copyright-holder.

## Funding

The two clinical cohorts received funding from 10.13039/501100004191Novo Nordisk A/s and the 10.13039/501100009708Novo Nordisk Foundation.

## Declaration of competing interest

The authors declare that they have no known competing financial interests or personal relationships that could have appeared to influence the work reported in this paper.

## Data Availability

Data will be made available on request.

## References

[1] World Health Organization (2024).

[2] Grüner Nielsen D., Andersen K., Søgaard Nielsen A. (2021 May). Consistency between self-reported alcohol consumption and biological markers among patients with alcohol use disorder - a systematic review. Neurosci. Biobehav. Rev..

[3] Perilli M., Toselli F., Franceschetto L. (2023 Jul 29). Phosphatidylethanol (PEth) in blood as a marker of unhealthy alcohol use: a systematic review with novel molecular insights. Int. J. Mol. Sci..

[4] Helander A., Hansson T. (2023 Oct). The alcohol biomarker phosphatidylethanol (PEth) - test performance and experiences from routine analysis and external quality assessment. Scand. J. Clin. Lab. Invest..

[5] Vanlerberghe B.T.K., Dumitrascu C., den Eede N.V. (2025 Apr 24). Phosphatidylethanol and ethyl glucuronide to categorize alcohol consumption in alcohol-related cirrhosis. JHEP Rep..

[6] Schomerus G., Leonhard A., Manthey J. (2022 Aug). The stigma of alcohol-related liver disease and its impact on healthcare. J. Hepatol..

[7] Helander A., Zheng Y. (2009). Molecular species of the alcohol biomarker phosphatidylethanol in human blood measured by LC-MS. Clin. Chem..

[8] Isaksson A., Walther L., Hansson T. (2011). Phosphatidylethanol in blood (B-PEth): a marker for alcohol use and abuse. Drug Test. Anal..

[9] Gnann H., Engelmann C., Skopp G. (2010). Identification of 48 homologues of phosphatidylethanol in blood by LC-ESI-MS/MS. Anal. Bioanal. Chem..

[10] Helander A., Böttcher M., Dahmen N. (2019). Elimination characteristics of the alcohol biomarker phosphatidylethanol (PEth) in blood during alcohol detoxification. Alcohol Alcohol.

[11] Fipps D.C., Meyer R., Woods J. (2024 Mar-Apr). Clinical utility and impact of phosphatidylethanol testing in liver transplantation evaluations. J Acad Consult Liaison Psychiatry.

[12] Vaz J., Nasr P., Helander A. (2025 Apr). Phosphatidylethanol levels distinguish steatotic liver disease subgroups and are associated with risk of major liver outcomes. J. Hepatol..

[13] Torp N., Bech K.T., Schnefeld H.L. (2025 Nov). Phosphatidylethanol and self-reported alcohol intake to subclassify individuals at risk of steatotic liver disease: an analysis of data from a prospective cohort study. Lancet Gastroenterol. Hepatol..

[14] Belyaeva E.V., Karacheva A.N., Bairova T.A. (2025 Jun). Phosphatidylethanol 16:0/18:1PEth as a biomarker of alcohol consumption during pregnancy. Bull. Exp. Biol. Med..

[15] Helander A., Hansson T. (2023 Jun 12). The alcohol biomarker phosphatidylethanol (PEth) - recommendations for use and interpretation of test results. Lakartidningen.

[16] Berg T., Eliassen E., Jørgenrud B. (2019 Jan). Determination of phosphatidylethanol 16:0/18:1 in whole blood by 96-well supported liquid extraction and UHPLC-MS/MS. J. Clin. Lab. Anal..

[17] Oppolzer D., Barroso M., Gallardo E. (2016). Bioanalytical procedures and developments in the determination of alcohol biomarkers in biological specimens. Bioanalysis.

[18] Ullah S., Helander A., Beck O. (2017 Aug 28). Identification and quantitation of phosphatidylethanols in oral fluid by liquid chromatography-tandem mass spectrometry. Clin. Chem. Lab. Med..

[19] Casati S., Ravelli A., Angeli I. (2019 Mar 29). An automated sample preparation approach for routine liquid chromatography tandem-mass spectrometry measurement of the alcohol biomarkers phosphatidylethanol 16:0/18:1, 16:0/16:0 and 18:1/18:1. Chromatogr A.

[20] Skråstad R.B., Spigset O., Aamo T.O. (2021). Stability of phosphatidylethanol 16:0/18:1 in freshly drawn, authentic samples from healthy volunteers. J. Anal. Toxicol..

[21] Cherrier M.M., Shireman L.M., Wicklander K. (2020). Relationshipof phosphatidylethanol biomarker to self-reported alcohol DrinkingPatterns in older and middle-age adults. Alcohol Clin. Exp. Res..

[22] Schröck A., Thierauf-Emberger A., Schürch S. (2016). Phosphatidylethanol (PEth) detected in blood for 3 to 12 days AfterSingle consumption of Alcohol—A drinking Study with 16 volun-teers. Int. J. Leg. Med..

[23] Beck O., Mellring M., Löwbeer C. (2021). Measurement of the Alcohol Biomarker phosphatidylethanol (PEth)in dried blood spots and venous blood-importance of inhibition ofPost- sampling Formation from ethanol. Anal. Bioanal. Chem..

